# Effects of threat cues on attentional shifting, disengagement and response slowing in anxious individuals

**DOI:** 10.1016/j.brat.2008.02.011

**Published:** 2008-05

**Authors:** Karin Mogg, Amanda Holmes, Matthew Garner, Brendan P. Bradley

**Affiliations:** School of Psychology, University of Southampton, Highfield, Southhampton SO17 1BJ, UK

**Keywords:** Anxiety, Attentional bias, Shift, Disengagement, Threat, Response slowing

## Abstract

According to cognitive models of anxiety, attentional biases for threat may cause or maintain anxiety states. Previous research using spatial cueing tasks has been interpreted in terms of difficulty in disengaging attention from threat in anxious individuals, as indicated by contrasts of response times (RTs) from threat cue versus neutral cue trials. However, on spatial cueing tasks, differences in RT between threat cue and neutral cue trials may stem from a slowing effect of threat on RT, as well as effects on allocation of visuospatial attention. The present study examined the effects of threat cues on both attentional cueing and response slowing. High and low anxious individuals completed a central cue task, which assessed threat-related response slowing, and a spatial cueing task, which assessed attentional biases for angry, happy and neutral faces. Results indicated that interpretation of the anxiety-related bias for threat depended on whether the effect of response slowing was taken into account. The study illustrates an important problem in using the modified spatial cueing task to assess components of threat-related attentional bias. As this experimental method may reflect both threat-related attentional cueing and response slowing effects, it cannot be assumed to provide pure measures of shift or disengagement components of attention bias.

## Introduction

Cognitive models of anxiety propose that biases in processing threat-related information—in particular, biases in selective attention and stimulus evaluation—may cause or maintain clinical anxiety (e.g., Beck & Emery, 1985; Eysenck, 1997; Mogg & Bradley, 1998; Williams, Watts, MacLeod, & Mathews, 1988, 1997). Attentional biases for threat have been investigated using various paradigms, including visual-probe (e.g., MacLeod, Mathews, & Tata, 1986) and spatial cueing tasks (e.g., Fox, Russo, Bowles, & Dutton, 2001). In the pictorial version of the visual-probe task (e.g., Mogg & Bradley, 1999), two pictures are presented together, one on each side of the screen (e.g., angry face and neutral face), and one of the pictures is replaced by a probe stimulus (e.g., dot or arrow). Participants are instructed to respond as quickly as possible to the probe. An index of attentional bias is calculated from response times (RTs) to probes. Several studies have indicated that anxious individuals are relatively faster to detect probes replacing threat cues than non-threat cues; which is indicative of an anxiety-related attentional bias for threat. This bias has been found in both clinical and non-clinical anxiety, using various stimuli, such as threat-related words and angry faces (e.g., MacLeod et al., 1986; Bradley, Mogg, Falla, & Hamilton, 1998; for reviews see Bar-Haim, Lamy, Pergamin, Bakermans-Kranenburg, & van IJzendoorn, 2007; Mogg & Bradley, 1998, 2004).

**Fig. 1:**
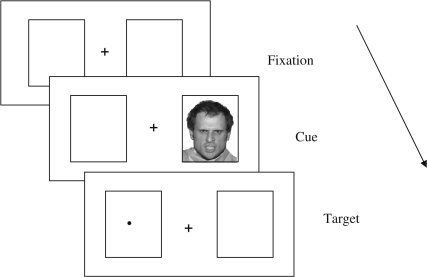
Example of a trial with an invalid threat cue; i.e., the threat cue (angry face) is in the opposite location to the probe (dot).

Distinctions have been made between different components of attention, such as shifting, engagement and disengagement functions (Posner & Petersen, 1990). Fox et al. (2001) used a modified spatial cueing paradigm (adapted from research by Posner, Inhoff, Friedrich, & Cohen, 1987) to investigate whether the anxiety-related attentional bias operates primarily in shift or disengage functions. The main trial events in a typical modified spatial cueing paradigm are illustrated in Fig. 1. On each trial, a single cue (e.g., angry or neutral face) is displayed on either the left or right side of the screen. The cue is replaced by a probe, which appears in either the same location (valid cue condition) or opposite location (invalid cue condition). Results from Fox et al. were interpreted to suggest that anxiety was associated with greater difficulty in *disengaging* attention from the spatial location of threat cues, as high state anxious individuals were relatively slower to respond to probes that were *invalidly* cued by threat, rather than neutral, stimuli. There was no evidence of an anxiety-related bias in *shifting* attention towards threat, relative to neutral cues, as there were no anxiety-related effects on *valid* trials (i.e., no difference in RT between valid threat and valid neutral cue conditions). However, Fox et al. noted that the task may have limited sensitivity to biases in attentional shifting, because RTs tend to be fast in the valid cue condition and ceiling effects might obscure any effect of threat on speed of orienting of attention. Nevertheless, the results from the invalid cue condition were consistent with the ‘delayed disengagement’ hypothesis, and further supportive evidence for this has been reported in other studies (e.g., Amir, Elias, Klumpp, & Przeworski, 2003; Fox et al., 2001; Fox, Russo, & Dutton, 2002; Koster, Crombez, Verschuere, & de Houwer, 2004; Yiend & Mathews, 2001).

However, the interpretation of results from this task has been questioned (Mogg & Bradley, 2008). The bias in *delayed disengagement* of attention from threat is estimated from invalid cue trials, by contrasting the RTs for *invalid threat* cues with RTs for *invalid neutral* cues. However, this contrast may be confounded by a response slowing effect of threat, which is conceptually distinct from effects of attentional cueing. Fox et al. (2001) noted that the delayed disengagement effect may reflect a ‘freezing’ response. Other theoretical views and empirical findings have also indicated that threat has a slowing or inhibition effect on motor responses, associated with temporary interruption of ongoing activity (e.g., Algom, Chajut, & Lev, 2004; Gray, 1982; Koster, Crombez, Verschuere, & De Houwer, 2004; McNaughton & Corr, 2004; Yiend & Mathews, 2001). However, if this is the case, interpretation of the results in terms of biases in shift or disengagement processes of visuospatial attention is called into question.

**Fig. 2:**
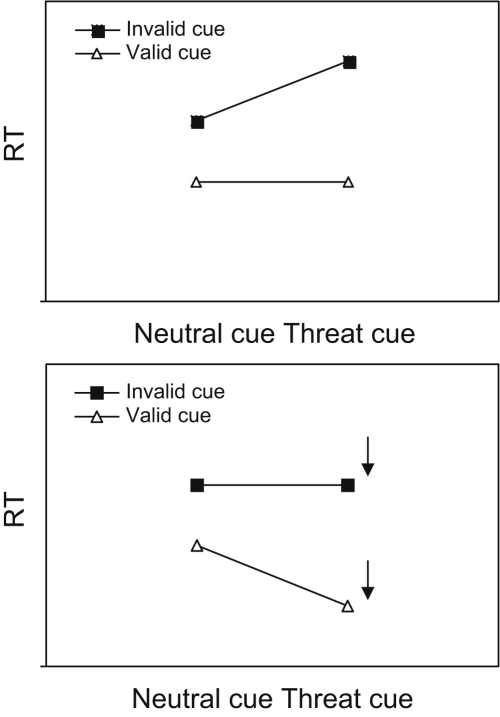
Hypothetical RT data from a spatial cueing experiment. Upper panel: RTs are slower on trials with invalid threat cues than invalid neutral cues (■), which seems consistent with a bias in delayed disengagement from threat. Lower panel: Slowing effect of threat cues on task performance has been subtracted from RTs on threat trials in both valid and invalid cue conditions (shown by ↓). RTs are now faster on trials with valid threat cues than valid neutral cues (Δ), which seems consistent with a bias in shifting attention to threat.

This confound is illustrated with hypothetical data in Fig. 2. The upper panel shows that RTs on valid cue trials are generally faster than those on invalid cue trials (i.e., the expected effect of cue validity on RT). There is also a cue valence×cue validity interaction. On *valid cue trials*, there is no difference in RTs between threat versus neutral cues, so there seems to be no evidence of a threat-related bias in attentional shifting. In contrast, on *invalid cue trials*, RTs are slower if the cues are threat related, rather than neutral, which seems to suggest a difficulty in disengaging attention from the threat location.

However, if threat cues have a slowing effect on RTs, as well as an attentional cueing effect, both should be taken into account when interpreting the results. In the interest of parsimony, it is assumed that these two hypothesised effects of threat cues on RT are additive. Moreover, if the RT slowing effect is estimated independently, it can then be subtracted from the mean RT in each of the valid threat and invalid threat cue conditions. Following correction of the data for the response slowing effect, there remains a cue valence×cue validity interaction (see lower panel of Fig. 2). However, RTs on valid threat cue trials are now faster than RTs on valid neutral cue trials, which is consistent with a bias in attentional shifting to threat. On the other hand, there is no longer any difference in RTs between invalid threat and invalid neutral cue trials, i.e., no evidence of a bias in attentional disengagement from threat. Thus, based on these assumptions, correction for a response slowing effect of threat on RTs reverses the interpretation of results, which now suggest a bias in attentional shifting to threat, but no bias in disengagement.

The main aims of the present study were to investigate whether these hypothetical effects operate under experimental conditions and to take account of response slowing when evaluating results from a spatial cueing task. The response slowing effect of threat was estimated on a central cue task, which was as similar as possible to the spatial cueing task (i.e., same cue and target stimuli, same exposure durations and response requirements); however, all stimuli were presented in the centre of the screen, so that no shifts of visuospatial attention were required. Consequently, any effect of threat cues, relative to neutral cues, on RT in the central cue task is likely to reflect a response slowing effect, which is independent of spatial cueing effects. In contrast, the RT data on the spatial cueing task are likely to reflect not only this response slowing effect (given the close similarities between the central cue and spatial cueing tasks), but also additional attentional effects associated with the spatial cueing requirements.

The main hypotheses were that anxiety-related attentional biases for threat should operate in both *shift* and *disengage* components of attention. That is, anxious individuals should show enhanced shifting of attention to threat (i.e., relatively faster RTs on valid threat than valid neutral cue trials) and also delayed disengagement from threat (i.e., relatively slower RTs on invalid threat than invalid neutral trials). These effects should be apparent even after correcting the RT data for any RT slowing effects of threat cues, which were estimated in the central cue task. The primary stimuli were angry (i.e., threat related) and neutral faces. A subsidiary issue was also addressed; as in Fox et al.'s (2001) studies, happy faces were included to examine whether similar attentional biases operate for positive information.

## Method

### Overview of design and hypotheses

The central cue task (which assessed the RT slowing effect of threat) was split into two blocks of trials: one block was presented before and one after the spatial cueing task. The central cue task had a 2×3×2 mixed factorial design, with anxiety (high and low) as a between-subjects independent variable (IV), and valence of face cue (angry, happy and neutral) and block (before vs. after spatial cueing task) as within-subject IVs. All face cues and targets were centrally located, so the task did not involve shifting or disengaging spatial attention. If threat has a response slowing effect, RTs should be slower on trials with threat than neutral cues.

The spatial cueing task used a 2×3×2 mixed factorial design, with anxiety (high and low) as a between-subjects IV, and cue valence (angry, happy and neutral) and cue validity (valid and invalid) as within-subject IVs. If anxious individuals have a bias in attentional shifting, they should show relatively faster RTs on valid angry face trials than valid neutral face trials, compared with low anxious participants. If anxious individuals have a bias in delayed disengagement, they should show relatively slower RTs on invalid angry face trials than invalid neutral face trials. These predicted effects should remain evident in analyses, which correct the RT data for the response slowing effect assessed on the central cue task. If anxiety-related attentional biases also operate for positive cues, corresponding effects should be found for trials with happy face cues, relative to neutral face cues.

### Participants

Participants were 51 students at the University of Southampton (13 male and 38 female; mean age of 20.8 years). The recruitment procedure was modelled on that used by Fox et al. (2001; Fox, personal communication): i.e., volunteers were screened prior to the study using the State-Trait Anxiety Inventory (STAI; Spielberger, Gorsuch, Lushene, Vagg, & Jacobs, 1983) and recruitment favoured those with either low or high trait anxiety scores in order to minimise the proportion of the sample with mid-range levels of state anxiety at the time of testing. Participants were subsequently allocated to two approximately equal-sized groups on the basis of their STAI-state anxiety scores in the test session, after excluding those with near-median scores: i.e., those scoring 35 or less were allocated to the low anxiety group (*n*=21), and a similar number who scored 38 or more were allocated to the high anxiety group (*n*=22; data from one high anxious participant were lost due to outliers, described later). Each participant had normal or corrected-to-normal vision and received either course credits or cash payment.

### Materials

The central cue task and spatial cueing task used the same face stimuli, which consisted of 48 colour photographs of 16 individuals, eight male and eight female, taken from the NimStim Set of Facial Expressions (available at http://www.macbrain.org/faces/; models were 01, 03, 06, 07, 09, 12, 16, 18, 23, 24, 29, 33, 34, 37, 40 and 42; each face with open mouth). The use of photographs of faces is similar to the cue stimuli used by Fox et al. (2001, Experiment 4). The faces portrayed three different emotional expressions of each individual: angry, happy and neutral. An additional four neutral faces (two male, two female) were used as practice and buffer items. Each face was presented within a grey box measuring 6.5 cm high×5 cm wide, superimposed on a black background. Targets were white up- and down-pointing arrows measuring 0.5 cm high, which appeared in the centre of the grey box. In the central cue task, the grey box remained at the centre of the screen throughout each trial. For the spatial cueing task, each face appeared within one of two grey boxes placed to the left and right of a white central fixation cross. The centres of the faces and grey boxes were 5 cm from fixation. The tasks were presented using Inquisit version 1.32 software. Testing was conducted in a dimly lit laboratory, with a computer screen at a viewing distance of 60 cm.

### Procedure

The order of experimental tasks was as follows: Central cue task (block 1); Spatial cueing task; Central cue task (block 2). The first block of trials on the central cue task consisted of four practice trials, followed by two buffer trials, and then 48 experimental trials. Each trial began with a central grey box for 1000 ms, which remained on the screen throughout the trial. A central face cue then appeared within the grey box for 200 ms, and 50 ms later a target (up or down arrow) was presented until a response was made or until 6 s had elapsed. Participants were instructed to press one of two vertically positioned buttons on a purpose-built response box, using the index finger of each hand, to indicate as quickly and as accurately as possible the type of target (i.e., they were instructed to press the upper key if the arrow pointed up). A tone was sounded during practice blocks if an error was made. There was a variable inter-trial interval (ITI) ranging from 500 to 1050 ms. At the end of the block of trials, the participant was presented with feedback on mean RT and percentage accuracy. There was an equal number of trials with each type of cue valence (angry, happy and neutral) and target, and the trials were presented in a new random order for each participant.

The spatial cueing task consisted of a block of 12 practice trials, followed by two buffer trials and 192 experimental trials. Each trial began with two grey boxes presented for 1000 ms to the left and right of a white central fixation cross (which remained on the screen throughout the trial). A face cue then appeared in the left or right grey box for 200 ms, and 50 ms later a target (up or down arrow) was presented until a response was made or until 6 s had elapsed. Participants were instructed to press one of two buttons on a purpose-built response box, using the index finger of each hand, to indicate as quickly and as accurately as possible the type of target. Thus, the study used a probe discrimination task (Mogg & Bradley, 1999). A tone sounded during practice trials if an error was made. There was a variable ITI ranging from 500 to 1050 ms.

Each face cue and target appeared equally often in left and right locations. As in Fox et al. (2001), the locations of the face cue and target were congruent on 75% of trials (i.e., the target appeared in the same spatial location as the face cue), and the locations of the face cue and target were incongruent on 25% of trials (i.e., the target appeared in the opposite location to the face cue). Thus, angry, happy and neutral faces each appeared 144 times in valid trials and 48 times in invalid trials. This resulted in four repetitions of each face image. At the start of the block, participants were asked to keep their gaze focused on the central fixation position throughout the task and were informed that the target was more likely to appear in the location of the face cue. At the end of the block, the participant was presented with feedback on their mean RT and percentage accuracy. The trials were presented in a new random order for each participant. The experimental block was presented in counterbalanced order with another block which manipulated the probability of occurrence of valid and invalid trials (i.e., 25% of trials were valid and 75% were invalid in the additional block), but which is not relevant to the hypotheses and provided no results of interest in relation to the research questions addressed here (further details are available on request from the authors).

Following the spatial cueing task, participants completed the second block of the central cue task. Next, they were given two other tasks lasting about 15 min, which are not presented here, as they focused on a different research question that was unrelated to anxiety-related attentional biases. At the end of the session, participants completed a series of questionnaires including the state and trait versions of the STAI.

### Preparation of RT data

RTs from incorrect trials were eliminated. Following inspection of the RT data with box-and-whisker plots, RTs which were less than 200 ms, or more than 1200 ms, and then those more than 3 SDs above each participant's mean were excluded in order to reduce the influence of outliers. Box-and-whisker plots indicated that one high anxious participant had unusually slow RTs, as indicated by outlying overall mean RTs and a number of outlying condition means on each task, so their data were excluded from the analyses [outliers were identified as values which were more than 1.5 box-lengths (i.e., inter-quartile range) above the 75th percentile]. The two groups did not differ significantly in the proportions of trials with incorrect responses or outliers, on any of the tasks (across both groups, mean error rates on the central cue task and spatial cueing task were .03 and .04, respectively; mean outlier rates were .003 and .01, respectively).

## Results

### Group characteristics

The low and high anxiety groups differed significantly in state anxiety (*M*=29.7, SD*=*4.3, vs. *M*=47.3, SD*=*7.1, *t*(41)=9.73, *p*<.01) and trait anxiety (*M*=35.4, SD*=*10.7, vs. *M*=45.8, SD*=*6.3, *t*(41)=3.90, *p*<.01), but not in age, (*M*=20.9, SD*=*3.0 vs. *M*=20.9, SD*=*2.9, *t*<1), or gender ratio (male: female ratio was 5:16 in the low anxiety group and 5:17 in high anxiety group, *χ*^2^=.01, NS).

### Central cue task

To estimate response slowing effects on the central cue task, an RT difference score was calculated for each participant by subtracting the mean RT on neutral cue trials from the mean RT on threat cue trials, so that positive values indicate a threat-induced slowing effect, and negative values indicate a threat-induced speeding effect. RT difference scores were similarly calculated for happy faces (i.e., mean RT on happy face cue trials minus mean RT on neutral cue trials); see Table 1 for mean RTs and difference scores in each condition. A 2×2×2 mixed design ANOVA of RT difference scores was carried out with anxiety (high and low), valence of face cue (angry and happy) and block (before vs. after spatial cueing task) as IVs. This showed a significant anxiety×cue valence interaction, *F*(1, 41)=4.49, *p*<.05, *η*_p_^2^=.10. To assess whether there was significant slowing in each group and valence condition, one-sample *t*-tests were used to compare the RT difference scores against a value of zero (0=no slowing). Threat cues had a significant slowing effect on RTs of high anxious individuals (*M*=9 ms, *t*(21)=2.39, *p*<.05, *d*=.51), but not low anxious individuals (*M*=2 ms, *t*(20)=0.75, *p*=.46, *d*=.17). Happy faces had a significant slowing effect on RTs of both high anxious (*M*=8 ms, *t*(21)=2.47, *p*<.05, *d*=.53) and low anxious (*M*=11 ms, *t*(20)=2.75, *p*<.05, *d*=.60) groups. The ANOVA showed no other significant results; e.g., the anxiety×cue valence interaction was not significantly influenced by block, *F*(1, 41)=1.46, *p*=.23.

### Spatial cueing task

A 2×3×2 ANOVA of RTs was carried out with anxiety (high, low), valence of face cue (angry, happy, neutral), and cue validity (valid, invalid) as IVs; see Table 2 for means and SDs. This showed a significant main effect of cue validity, *F*(1, 41)=246, *p*<.001, *η*_p_^2^=.86, as RTs were faster on valid trials than invalid trials (422 vs. 511 ms). This was qualified by a significant anxiety group×cue valence×cue validity interaction, *F*(2, 82)=4.92, *p*=.01, *η*_p_^2^=.11. There were no other significant results. To break down the 3-way interaction and test our hypotheses, a series of 2×2 ANOVAs were carried out to examine the effect of each type of emotional cue, relative to neutral cues, in the valid and invalid cue conditions separately.

(i) *Valid threat cues*. To examine whether there was an anxiety-related bias for threat cues in attentional shift processes, an ANOVA of RTs from valid trials was carried out with anxiety and cue valence (angry and neutral) as IVs. This showed no significant results (anxiety×cue valence interaction: *F*(1, 41)=2.02, *p*=.16).
(ii) *Invalid threat cues*. To examine whether there was a bias for threat in disengagement processes, an ANOVA of RTs from invalid trials was carried out with anxiety and cue valence (angry and neutral) as IVs. This showed only a significant anxiety×cue valence interaction, *F*(1, 41)=5.80, *p*<.05, *η*_p_^2^=.12: the high anxiety group was relatively slower to respond to probes with invalid threat cues than invalid neutral cues (508 vs. 488 ms, respectively, *p*<.05), compared with the low anxiety group (523 vs. 531 ms, respectively).
(iii) *Valid happy cues*. An ANOVA of RTs from valid trials, with anxiety and cue valence (happy and neutral) as IVs, showed no significant results.
(iv) *Invalid happy cues*. An ANOVA of RTs from invalid trials, with anxiety and cue valence (happy and neutral) as IVs, showed a significant main effect of anxiety, *F*(1, 41)=4.29, *p*<.05, as the low anxious group had a slower mean RT than the high anxious group (526 vs. 493 ms), but no other significant results.

### Spatial cueing task: data corrected for estimated RT slowing effect of emotional cues

The RT data from threat trials in the spatial cueing task were adjusted by subtracting the slowing (or speeding) effect of threat cues on RTs (i.e., the RT difference score which had been assessed on the central cue task) from the corresponding mean RT in each cue condition for each participant. E.g., if a person was on average 10 ms slower to respond on trials with angry than neutral cues on the central cue task (which reflects a 10 ms slowing effect of threat on RT, independent of location-based spatial cueing effects), this value was subtracted from their mean RT from trials with angry face cues in each condition (valid and invalid) of the spatial cueing task. The RT data from trials with happy faces were corrected in a similar manner: i.e., for each participant, the RT difference score for happy faces, relative to neutral faces (estimated on the central cue task), was subtracted from the mean RT from valid happy trials, and also from the mean RT from invalid happy trials, in the spatial cueing task.

A 2×3×2 ANOVA of RTs was carried out of the corrected RT data with anxiety group (high and low), valence of face cue (angry, happy and neutral), and cue validity (valid and invalid) as IVs (see Table 2 for means). This showed significant main effects of cue validity, *F*(1, 41)=246, *p*<.001, *η*_p_^2^=.86, and cue valence, *F*(2, 82)=3.46, *p*<.05, *η*_p_^2^=.08, which were qualified by a significant anxiety group×cue valence×cue validity interaction, *F*(2, 82)=4.92, *p*=.01, *η*_p_^2^=.11. There were no other significant results. To breakdown the 3-way interaction and test our hypotheses, a series of ANOVAs were carried out on the corrected RT data, similar to those described earlier.

(i) *Valid threat cues*. An ANOVA of RTs from valid trials, with anxiety and cue valence (angry and neutral) as IVs, showed a significant anxiety×cue valence interaction, *F*(1, 41)=4.49, *p*<.05, *η*_p_^2^=.10. The high anxiety group was faster to respond to probes with valid threat cues than valid neutral cues (405 vs. 419 ms, respectively, *p*<.05), compared with the low anxiety group (427 vs. 425 ms, respectively).
(ii) *Invalid threat cues*. An ANOVA of RTs from invalid trials, with anxiety and cue valence (angry and neutral) as IVs, showed no significant results (anxiety×cue valence interaction: *F*(1, 41)=2.80, *p*=.10).
(iii) *Valid happy cues*. An ANOVA of RTs from valid trials, with anxiety and cue valence (happy and neutral) as IVs, showed a significant main effect of face type, *F*(1, 41)=8.27, *p*<.05, *η*_p_^2^=.17, which was not significantly influenced by anxiety, *F*<1. Both groups were faster on valid happy face trials (412 ms) than on valid neutral trials (422 ms).
(iv) *Invalid happy cues*. An ANOVA of RTs from invalid trials, with anxiety and cue valence (happy and neutral) as IVs, showed no significant results.

### Spatial cueing task: attentional bias scores

A general measure of attentional bias for threat cues can also be obtained from the spatial cueing task, which is comparable with the attentional bias index obtained from the visual-probe task (e.g., Bradley et al., 1998) and which does not attempt to assess separately the component processes of shift and disengagement. This measure summarises the interaction effect of cue validity (valid and invalid)×cue valence (threat and neutral) on RTs, as it is the difference between the cueing effect of threat cues and the cueing effect of neutral cues: i.e., attentional bias score=(mean RT from invalid threat trials−mean RT from valid threat trials)−(mean RT from invalid neutral trials−mean RT from valid neutral trials). Positive values indicate an attentional bias for threat, relative to neutral, cues. The high anxious group had greater attentional bias scores for threat cues than the low anxious group: mean threat bias scores were 24.6 ms (SD=46.9) vs. −12.2 ms (SD=39.1), respectively, *t*(41)=2.79, *p*<.01. A notable feature of these scores is that they are unaffected by the correction for RT slowing, described earlier, as the correction (subtraction) is applied to both valid and invalid conditions. Thus, attentional bias scores (difference in RTs between invalid and valid trials) remain unchanged; 24.6 vs. −12.2 ms, respectively, *t*(41)=2.79, *p*<.01. An attentional bias index was also calculated for happy face cues. The high anxious group had greater attentional bias scores for happy cues than the low anxious group: mean happy bias scores were 12.3 ms (SD=26.9) vs. −12.7 ms (SD=38.9), respectively, *t*(41)=2.46, *p*<.05.

## Discussion

The results from the initial analyses of RT data from the spatial cueing task appeared to be consistent with the hypothesis that anxiety is associated with a greater difficulty in disengaging attention from the spatial location of threat cues. That is, high anxious individuals showed relatively slower RTs on trials with invalid threat cues than invalid neutral cues, compared with low anxious participants. These initial analyses showed no evidence of a corresponding bias in the shift component of attention (i.e., the anxiety×cue valence interaction was not significant for valid trials). These findings seem compatible with those of Fox et al. (2001) and Yiend and Mathews (2001).

However, these analyses took no account of the possible existence of a RT slowing effect of threat cues, which was confirmed on the central cue task. This showed that threat cues significantly slowed RT in high, but not low, anxious individuals, whereas happy cues slowed RT in both high and low anxious groups. These RT slowing effects cannot be attributed to spatial cueing effects, as there was no requirement for participants to shift or disengage spatial attention on the central cue task. When the data from the spatial cueing task were re-analysed, after using a subtractive procedure to adjust for the RT slowing effect of emotional cues (which had been estimated on the central cue task), a different pattern of results emerged. These were now consistent with anxiety being associated with an increased bias to shift attention towards threat cues (i.e., high anxious individuals showed relatively faster RTs on trials with valid threat cues than valid neutral cues), but no evidence of a bias in disengagement processes. Thus, the results from the uncorrected and corrected RT data give rise to contradictory conclusions regarding the component processes, which underlie attentional biases in anxiety.

The interpretation of results from the spatial cueing task depends on the accuracy of the estimate of RT slowing from the central cue task. The central cue task did not have any requirement to shift or disengage spatial attention from the location of the cue, and was designed to resemble the spatial cueing task as closely as possible, with the exception of removing the spatial cueing component. However, there were other minor methodological differences between the two tasks. For example, the fixation cross remained displayed throughout each spatial cueing trial, but not during central cue trials (the purpose of this stimulus arrangement was to encourage a central focus of attention in both tasks, while minimising the likelihood of any shifts of spatial attention occurring during the central cue task). Thus, the estimate of RT slowing on the central cue task may not have been a perfect estimate of the RT slowing effect occurring on the spatial cueing task. In addition, for the sake of parsimony, we assumed here that, on the spatial cueing task, the separate effects of threat on RT slowing and spatial cueing (either shifting or disengaging attention from the spatial location of the cue) were additive, and it would clearly be desirable to have evidence to justify this assumption. Thus, it would seem advisable to refine and improve this methodology in future research into shift and disengage components of visuospatial attentional biases.

Despite this note of caution, the present findings are important in showing that attempting to control for RT slowing effects (which did not involve shifts in visuospatial attention) can fundamentally alter the interpretation of results from the spatial cueing task. Consequently, although the present results indicated an attentional bias to threat in anxious individuals (as reflected by attentional bias scores), their interpretation in terms of separate components of either shift or disengagement processes is uncertain. There was evidence of an anxiety-related bias in disengaging attention from threat in the uncorrected RT data, and evidence of an anxiety-related bias in shifting attention to threat after correcting for RT slowing. Clearly, this issue can only be resolved by further investigation.

Various mechanisms might be responsible for a response slowing effect of threat cues. For example, it may be due to a behavioural inhibition system (BIS), which Gray (1982) proposed is activated by novel or threat stimuli; the BIS then interrupts ongoing information processing and behavioural responses, and triggers increased arousal and vigilance, which facilitate responding to the potential threat (see also McNaughton & Corr, 2004). Another possibility is that RT slowing reflects a distracting influence of task-irrelevant thoughts elicited by emotional cues, which compete for processing resources. The present results suggested that the RT slowing effect on the central cue task was an interactive function of the valence of the face cues and participants’ anxiety level. That is, low anxious individuals only showed RT slowing in response to happy faces, whereas high anxious individuals showed RT slowing effects of both angry and happy faces, relative to neutral faces. This does not seem easily explained by a simple BIS account and may suggest that more than one mechanism may be contributing to these effects.

RT slowing effects are also relevant to the interpretation of results from other tasks assessing attentional biases. Such effects may contribute to the anxiety-related interference effect of threat cues on the modified Stroop task (Algom et al., 2004), as well as other tasks used to investigate disengagement biases. For example, one method has involved the central presentation of a cue (e.g., threat or neutral word; or fearful or neutral face) and, after a short delay (e.g., 600 ms), a target probe (e.g., asterisk or letter) appears beside the central cue and participants respond as quickly as possible to the probe (Fox et al., 2001, Experiment 5; Georgiou et al., 2005). The latter studies indicated that anxious individuals had slower RTs when the central cue was threat-related rather than neutral. One interpretation of these findings is in terms of an anxiety-related difficulty in disengaging attention from the central threat cue, but they might also be accounted for by a greater slowing effect of threat cues on motor responses in anxious individuals. This task has also been used by Bar-Haim, Lamy, and Glickman (2005) who concurrently assessed event-related brain potentials (ERPs) to the central face cues. The RT data failed to replicate previous findings (Fox et al., 2001; Georgiou et al., 2005), but the ERP data indicated that high anxious individuals showed a greater P2 amplitude to angry faces than low anxious individuals. Bar-Haim et al. (2005) suggested that the P2 data may reflect enhanced allocation of attentional resources to threat cues in anxiety. Thus, ERP methodology may provide a useful tool for assessing attentional biases, even when such biases are not revealed by manual RT measures.

The visual-probe task provides a widely used index of attentional bias (e.g., Mogg & Bradley, 1999, for discussion of methodological issues), which seems less susceptible to interpretive problems associated with RT slowing effects, because a threat stimulus is presented on each critical trial (the conventional attentional bias index involves contrasting two main cueing conditions; i.e., whether the probe is in the same or opposite location to the threat cue on trials in which both threat and neutral cues are presented). Thus, any RT slowing effect of threat is likely to be constant across all critical trials. A limitation of the visual-probe task is that it does not provide separate measures of shift or disengagement processes. Although some recent studies have attempted to use this task to examine shift and disengagement biases (e.g., Koster et al., 2004; Salemink, van den Hout, & Kindt, 2007), this approach is susceptible to the same problems as noted earlier for the spatial cueing task. Specifically, assessment of a disengagement bias relied on subtracting the mean RT on trials with neutral stimuli (i.e., no threat stimulus present) from the mean RT on trials where the probe replaced a neutral stimulus that was paired with a threat stimulus. Thus, a difference in RT between these conditions could reflect two separate effects, i.e., a slowing effect of threat on RT and a difficulty in disengaging attention from the threat stimulus. Consequently, this difference in RT cannot be assumed to provide a pure index of disengagement processes. Nevertheless, the visual-probe task has been used to examine the role of awareness and different functional components of attention, such as initial orienting (which includes disengage/shift operations) versus maintenance of attention (e.g., reviews by Bar-Haim et al., 2007; Mogg & Bradley, 1998, 2004, 2008), and has been complemented by concurrent eye-tracking (e.g., Bradley, Mogg, & Millar, 2000; Garner, Mogg, & Bradley, 2006; Mogg, Millar, & Bradley, 2000).

The present study raised other issues that were subsidiary to the main hypotheses. First, the supplementary analyses indicated that the general index of attentional bias was unaffected by the RT slowing effect of threat. Thus, the modified spatial cueing task can provide a general index of attentional bias for threat, even though it does not provide separate measures of biases in shift or disengagement processes. Second, the analyses of attentional bias scores suggested that, in comparison with the low anxious group, the high anxious group had a greater attentional bias not only for angry faces, but also for happy faces. The components of the bias for happy faces were unclear because the primary analyses of RTs for happy versus neutral faces showed non-significant results within each of the valid and invalid cue conditions. Some previous studies have also indicated anxiety-related attentional biases for positive stimuli (e.g., Bradley, Mogg, White, Groom, & de Bono, 1999; Garner et al., 2006; Martin, Williams, & Clark, 1991), although others have suggested that anxiety-related biases are specific to threat, rather than positive, stimuli (e.g., Bradley et al., 1998, 2000; Fox et al., 2001; Mogg, Philippot, & Bradley, 2004). Thus, the determinants of positive processing biases remain unclear and require further investigation (e.g., Becker, Rinck, Margraf, & Roth, 2001; Williams, Mathews, & MacLeod, 1996).

In conclusion, given the problem in interpreting the results from the modified spatial cueing task, we propose that the ‘delayed disengagement’ hypothesis remains untested. It seems inadvisable for researchers to continue using this task in order to assess threat-related biases in shift and disengagement processes separately. This conclusion arises from reasoned argument based on prior theory and empirical findings, and does not rely solely on the experimental data presented here. That is, differences in RT between threat cue and neutral cue trials may result from two separate effects: (i) a slowing effect of threat cues on motor responses, which may reflect a temporary inhibition of ongoing activity, and (ii) an effect of threat stimuli on allocation of visuospatial attention. Evidence of the former effect (i.e., threat-related response slowing) is provided not only by the data from the central cue task reported here, but also other sources (e.g., Algom et al., 2004; Yiend & Mathews, 2001). Hence, if it is accepted that emotion-related response slowing effects can occur independently of visuospatial attention cueing effects, it follows that contrasts of RT data between threat cue and neutral cue trials cannot be assumed to provide pure measures of either shift or disengage components of attentional biases. It therefore remains an important and positive challenge for investigators to develop paradigms that will clarify further the component processes of attentional biases in anxiety.

## Figures and Tables

**Table 1:** Mean RTs and RT difference scores in ms (SDs in parentheses) for low and high state anxiety groups in each condition of the central cue task

	Low anxiety		High anxiety	
	*M*	SD	*M*	SD
*RTs to probes*				
Block 1				
Angry	377	(41)	379	(47)
Happy	383	(36)	382	(44)
Neutral	374	(38)	371	(47)
Block 2				
Angry	384	(46)	382	(52)
Happy	396	(33)	376	(57)
Neutral	382	(39)	371	(51)
Averaged across both blocks				
Angry	381	(37)	380	(47)
Happy	390	(31)	379	(46)
Neutral	378	(32)	371	(46)
*RT difference scores—averaged across both blocks*				
Angry	2	(15)	9	(18)
Happy	11	(19)	8	(15)

**Table 2:** Mean RTs in ms (SDs in parentheses) for low and high state anxiety groups in each condition of the spatial cueing task

	Low anxiety		High anxiety	
	*M*	SD	*M*	SD
*Spatial cueing task*				
Valid trial				
Angry	429	(45)	415	(41)
Happy	427	(38)	416	(40)
Neutral	425	(35)	419	(43)
Invalid trials				
Angry	523	(68)	508	(57)
Happy	521	(61)	497	(42)
Neutral	531	(62)	488	(54)
*Spatial cueing task—corrected for RT slowing effect*				
Valid trials				
Angry	427	(42)	405	(47)
Happy	415	(43)	408	(41)
Neutral	425	(35)	419	(43)
Invalid trials				
Angry	521	(66)	499	(62)
Happy	509	(64)	489	(47)
Neutral	531	(62)	488	(54)
